# 

**Published:** 1859-07

**Authors:** 


### NOTICE.

All communications relating to the Editorial Department, Journals, Books for Review, etc., must be directed to AUSTIN FLINT, JR., M. D., EDITOR, NEW YORK CITY.

All communications relating to the Business Department, Advertising, Remittances, etc., must be directed to A. I. MATHEWS, PUBLISHER, BUFFALO, N. Y.

☞ Correspondents of Dr. Austin Flint, Sr., will please direct to Gramercy Park House, New York City.

